# Case of Great Hypertrophy of the Heart and Open Foramen Ovale, without Cyanosis

**Published:** 1844-06-15

**Authors:** J. D. White, James Bryan

**Affiliations:** Philad’a.; Philad’a.


### THE MEDICAL EXAMINER,

### And Record of Medical Science.

Vol. VII.] PHILADELPHIA, SATURDAY, JUNE 15, 1844. [No. 12.

### CASE OF GREAT HYPERTROPHY OF THE HEART AND OPEN FORAMEN OVALE, WITHOUT CYANOSIS.

By Drs. J. D. White and James Bryan, of Philad'a.

(Communicated by Prof. Dunglison.)

*January* 3, 1843.—*Post Mortem of Josephine, aged* 4 *months and* 15 *days.*

General external appearance not much changed from that of health; very little emaciation; no discoloration of the skin; hair black; skin fair.

On raising the sternum, the heart was found in its usual position. The right lung was healthy, and in a normal condition; the left lung, with the exception of a small portion of the upper lobe, was very much contracted, of a chocolate colour, and lying close along the spine.

On opening the pericardium, the liquor pericardii appeared in unusual quantity—the heart very much enlarged, and of a deep red colour. The vessels on the surface were increased in size, and distended with blood; the surfaces of the heart and pericardium smooth and free from any appearances of inflammatory action.

The parietes when opened were found to measure in the left ventricle one inch and two-tenths; in the right ventricle six-tenths of an inch, and the septum half an inch in thickness. The auricles appeared small and disproportioned to the ventricles, and the *foramen ovale* entirely *open*, so that the little finger passed through it easily.

The liver was considerably enlarged, especially the right lobe, and of a very dark colour.

In this case, no appearances of disease exhibited themselves, until about two weeks after birth, when restlessness on lying down, particularly at night, and on the right side, occurred, which was relieved by turning the child over on the *left* side, or raising it to a *sitting* posture. This continued, slightly increasing, the general health in other respects good, until within five days of its death, when severe symptoms of catarrh accompanied with some difficulty of breathing supervened, and terminated fatally on the third of January, 1843. The catarrh was treated by nauseants and expectorants without any obviously good effect.

Beside the extraordinary size of the heart, the foregoing case affords additional evidence that the non-closure of the foramen ovale does not necessarily give rise to cyanosis. We have now abundant testimony that cyanosis may occur without an open state of the foramen ovale, and that that opening may remain unclosed without the blue disease: so that the testimony upon the subject is both positive and negative, and must therefore be regarded as conclusive against the opinion long entertained on that point.

In nearly every case in which cyanosis has existed, malformation of the heart, lungs, aorta, or pulmonary arteries, has been observed on dissection; and the patulous state of the foramen, when present, seems to have been an incident, rather than the cause, of that disease. A full statement of the evidence on this subject will be found in Dr. Condie's excellent work on *Diseases of Children*, page 534, and also in Professor Dunglison's *Practice of Medicine*, second edition, vol. i. p. 486.—Ed.

